# PIP2, An Auxin Induced Plant Peptide Hormone Regulates Root and Hypocotyl Elongation in *Arabidopsis*

**DOI:** 10.3389/fpls.2021.646736

**Published:** 2021-05-14

**Authors:** Saddam Hussain, Wei Wang, Sajjad Ahmed, Xutong Wang, Adnan, Yuxin Cheng, Chen Wang, Yating Wang, Na Zhang, Hainan Tian, Siyu Chen, Xiaojun Hu, Tianya Wang, Shucai Wang

**Affiliations:** ^1^Laboratory of Plant Molecular Genetics & Crop Gene Editing, School of Life Sciences, Linyi University, Linyi, China; ^2^Key Laboratory of Molecular Epigenetics of MOE, Northeast Normal University, Changchun, China

**Keywords:** auxin, peptide hormone, PIP2, PIP3, root elongation, *Arabidopsis*

## Abstract

Auxin is one of the traditional plant hormones, whereas peptide hormones are peptides with hormone activities. Both auxin and plant peptide hormones regulate multiple aspects of plant growth and development, and there are cross-talks between auxin and plant peptide hormones. PAMP-INDUCED SECRETED PEPTIDES (PIPs) and PIP-LIKEs (PIPLs) are a new family of plant peptide hormone, and PIPL3/TARGET OF LBD SIXTEEN 2 (TOLS2) has been shown to regulate lateral root formation in *Arabidopsis*. We report here the identification of *PIP2* as an auxin response gene, and we found it plays a role in regulating root and hypocotyl development in *Arabidopsis*. By using quantitative RT-PCR, we found that the expression of *PIP2* but not *PIP1* and *PIP3* was induced by auxin, and auxin induced expression of *PIP2* was reduced in *nph4-1* and *arf19-4*, the lost-of-function mutants of *Auxin Response Factor 7* (*ARF7*) and *ARF19*, respectively. By generating and characterizing overexpressing transgenic lines and gene edited mutants for *PIP2*, we found that root length in the *PIP2* overexpression plant seedlings was slightly shorter when compared with that in the Col wild type plants, but root length of the *pip2* mutant seedlings remained largely unchanged. For comparison, we also generated overexpressing transgenic lines and gene edited mutants for *PIP3*, as well as *pip2 pip3* double mutants. Surprisingly, we found that root length in the *PIP3* overexpression plant seedlings is shorter than that of the *PIP2* overexpression plant seedlings, and the *pip3* mutant seedlings also produced short roots. However, root length in the *pip2 pip3* double mutant seedlings is largely similar to that in the *pip3* single mutant seedlings. On the other hand, hypocotyl elongation assays indicate that only the *35S:PIP2* transgenic plant seedlings produced longer hypocotyls when compared with the Col wild type seedlings. Further analysis indicates that PIP2 promotes cell division as well as cell elongation in hypocotyls. Taken together, our results suggest that *PIP2* is an auxin response gene, and PIP2 plays a role in regulating root and hypocotyl elongation in *Arabidopsis* likely *via* regulating cell division and cell elongation.

## Introduction

The plant hormone auxin regulates multiple aspects of plant growth and development largely by activating the expression of auxin response genes (Davies, 1995; Chapman and Estelle, 2009). The activation of auxin response genes is mainly regulated by the TRANSPORT INHIBITOR RESPONSE 1 (TIR1) auxin receptor (Dharmasiri et al., 2005; Kepinski and Leyser, 2005), and two different families of transcription factors, i.e., the AUXIN RESPONSE FACTOR (ARF) family and the AUXIN (Aux)/INDOLE-3-ACETIC ACID (IAA) protein family (Guilfoyle et al., 1998; Reed, 2001; Guilfoyle and Hagen, 2007). Five of the ARFs, including ARF5, ARF6, ARF7, ARF8, and ARF19 function as transcription activators and are able to activate the expression of auxin response genes (Tiwari et al., 2003; Wang et al., 2005). However, when the level of cellular auxin is low, Aux/IAA proteins, the transcription repressors in auxin signaling (Tiwari et al., 2004), can form dimmers with ARF activators and inhibit their activities (Tiwari et al., 2003). When the level of cellular auxin is elevated, auxin are able to bind and activate the TIR1 auxin receptor, leading to degradation of Aux/IAA proteins *via* 26S proteasome, therefore release the inhibition of Aux/IAA proteins on ARF activators, resulting in activation of auxin response genes (Guilfoyle and Hagen, 2007; Tan et al., 2007; Hayashi, 2012).

So far several different gene families such as *Aux*/*IAAs*, *GRETCHEN HAGENs* (*GH3s*), and *SMALL AUXIN-UP RNAs* (*SAURs*; Hagen and Guilfoyle, 2002), and some other genes such as *ASYMMETRIC LEAVES2-LIKE*/*LATERAL ORGAN BOUNDARIES DOMAIN* (*ASL*/*LBD*), *PACLOBUTRAZOL RESISTANCE 6* (*PRE6*) and *LATERAL ROOT PRIMORDIUM1* (*LRP1*; Lee et al., 2009; Coudert et al., 2013; Zheng et al., 2017; Singh et al., 2020), have been identified as auxin response genes. However, considering that auxin is involved in the regulation of almost all the aspects of plant growth and development, large numbers of auxin response genes should still remain unidentified (Kieffer et al., 2010). On the other hand, exploration of the functions of the auxin response genes is still on going, as an example, the *SAURs* were identified as an auxin response gene family about 25 years ago (Gil et al., 1994), yet it is only in recent years that SAURs have been identified to regulate several different aspects of plant growth and development, such as cell expansion (Spartz et al., 2012; Kong et al., 2013; Qiu, et al., 2020), pollen tube growth (He et al., 2018), apical hook development (Kathare et al., 2018), hypocotyl and stamen filament elongation (Chae et al., 2012), and leaf senescence (Hou et al., 2013; Wen et al., 2020).

Peptide hormones are peptides with hormone activities in animal, bacteria and yeast (Edlund and Jessell, 1999). The first plant peptide hormone, systemin, was identified about 30 years ago (Pearce et al., 1991), and more than 20 different types of plant peptide hormones have been identified since then (Hirakawa et al., 2017; Hirakawa and Sawa, 2019). Plant peptide hormones are also involved in the regulation of different aspects of plant growth and development. As examples, CLAVATA3/ENDOSPERM SURROUNDING REGIONs (CLEs) regulate the maintains of shoot and root apical meristem (Kinoshita et al., 2007; Jun et al., 2010; Katsir et al., 2011; Guo et al., 2015), POLARIS (PLS), AUXIN-RESPONSICE ENDOFENOUS POLYPEPTIDE 1(AREP1) and GROWTH FACTOR/CLE LIKE/GOLVEN (RGF/CLEL/GLV) regulate root growth (Casson et al., 2002; Matsuzaki et al., 2010; Meng et al., 2012a; Fernandez et al., 2013; Yang et al., 2014), RGF/CLEL/GLV regulates lateral root formation (Matsuzaki et al., 2010; Meng et al., 2012a; Fernandez et al., 2013), PLS regulates vascular development (Casson et al., 2002), EPIDERMAL PATTERNING FACTORs (EPFs) regulate stomata development (Hara et al., 2007; Hunt and Gray, 2009; Sugano et al., 2010), DEVIL (DVL1) and ROTUNDIFOLIA4 (ROT4) regulate leaf and fruit development (Narita et al., 2004; Wen et al., 2004), and INFLORESCENCE DEFICIENT IN ABSCISSION LIKEs (IDLs) regulate floral organ abscission (Butenko et al., 2003; Cho et al., 2008; Stenvik et al., 2008).

At least some of the aspects of plant growth and development are regulate by both auxin response genes and plant peptide hormones. For example, both the plant peptide hormones PLS, AREP1 and RGF/CLEL/GLV and some Aux/IAA proteins such as IAA9 are able to regulate root growth (Casson et al., 2002; Matsuzaki et al., 2010; Meng et al., 2012a; Fernandez et al., 2013; Yang et al., 2014), and auxin is involved in CLE regulated vascular proliferation (Whitford et al., 2008). Some other experiments have also indicated that there are cross-talk between auxin and some of plant peptide hormones. For example, the expression of *PLS*, *AREP1* and *RGF*/*CLEL*/*GLV* genes are induced by auxin, whereas PLS and RGF/CLEL/GLV peptides are able to regulate auxin transport (Casson et al., 2002; Chilley et al., 2006; Meng et al., 2012b; Whitford et al., 2012; Yang et al., 2014).

PAMP-INDUCED SECRETED PEPTIDES (PIPs) and PIP-LIKEs (PIPLs) are a new family of plant peptide hormone identified in *Arabidopsis* in recent years (Hou et al., 2014; Vie et al., 2015). Both PIP and PIPL propeptides have an N-terminal signal peptide and a C-terminal SGPS motif, which is part of the biologically active peptides, with an exception of PIP2 and PIP3 prepropeptides, which have two SGPS motifs (Hou et al., 2014; Vie et al., 2015). The PIP peptides including PIP1, PIP2 and PIP3 have been shown to modulate immunity (Hou et al., 2014; Najafi et al., 2020), and the expression of several *PIPs* and *PIPLs* family genes is induced by biotic and/or abiotic stresses (Hou et al., 2014; Vie et al., 2015). On the other hand, it has been reported that the PIPL3/TARGET OF LBD SIXTEEN 2(TOLS2) is able to regulate lateral root formation (Toyokura, et al., 2019). Here, we report the identification of *PIP2* as an auxin response gene, and we found that PIP2 is involved in the regulation of root and hypocotyl development in *Arabidopsis*.

## Materials and Methods

### Plant Materials and Growth Conditions

The Columbia-0 (Col) ecotype *Arabidopsis* (*Arabidopsis thaliana*) was used as wild type for plant transformation and auxin response analysis of the *PIP* genes, and as a control for root length, hypocotyl length, cell number and cell length analysis. The *nph4-1* and *arf19-4* mutants are in the Col wild type background (Harper et al., 2000; Wang et al., 2005). The *35S:PIP2* and *35S:PIP3* overexpress plants were generated by transforming Col wild type plants, and the *pip2* and *pip3* single and the *pip2 pip3* double mutants were obtained by editing *PIP2* and *PIP3* genes in the Col wild type plants *via* CRISPR/Cas9 gene editing techniques.

For plant transformation, the Col wild type seeds were sown directly into the soil pots and grown in a growth chamber. To obtain seedlings for auxin treatment and phenotypic analysis, seeds of the Col wild type, the *nph4-1*, *arf19-4*, *pip2*, *pip3*, and *pip2 pip3* mutants, and the *35S:PIP2* and *35S:PIP3* overexpress plants were surface sterilized with 25% (v/v) bleach for 10 min, washed with sterile deionized water for four times, and then sown on 1/2 Murashige and Skoog (MS) petri plates, containing vitamins (Plant Media), 1% (w/v) sucrose, pH 5.8, and solidified with 0.6% (w/v) phytoagar (Plant Media). The plates were then kept in 4°C for 2 days, and then moved to a growth chamber.

The growth condition in the growth chamber was set as 23°C temperature, 60% relative humidity conditions, and photon density set at ~120 μmol m^−2^ s ^−1^ under a 16 h light/8 h dark photoperiod unless indicated otherwise.

### Auxin Treatment, RNA Isolation, and Quantitative RT-PCR

To examine the expression of *PIP2* and *PIP3* in response to auxin, 10-day-old Col wild type seedlings were transferred to petri plates containing 10 μM IAA and shaked on a shaker in dark for 4 h. To examine auxin regulated epression of *PIP2* and *IAA19* in *nph4-1* and *arf19-4* mutants, 10-day-old Col wild type, and *nph4-1* and *arf19-4* mutant seedlings were treated with 10 μM IAA for 4 h. Seedlings were collected, total RNA was isolated, cDNA was synthesized as described previously (Wang et al., 2015a), and used to detect the expression of *PIP2*, *PIP3* and *IAA19* with a process described previously (Wang et al., 2015b), and the expression of *ACTIN2* (*ACT2*) gene was used as an internal control. The primers used for quantitative RT-PCR (qRT-PCR) analysis of *IAA19* and *ACT2* have been described previously (Liu et al., 2015; Wang et al., 2015a,b), and analyzed by using delta delta method (∆∆Ct). The primers used for qRT-PCR analysis of *PIP2* and *PIP3* were 5'-GGAGAAGTTCGTGGCTAGTTTAT-3' and 5'-CTTCCTGTCCACGACCTTATG-3', 5'-AGAGAACCTCGTGGCTAAGT-3' and 5'-GGGACCTGAATGCTTACCATATT-3' respectively.

### Constructs

To generate *pPZP-35S:PIP2* and *pPZP-35S:PIP3* constructs for plant transformation, the full length open-reading frame (ORF) sequences of *PIP2* and *PIP3* were amplified and inserted, respectively into the *pUC19* vector with an N-terminal HA tag using NdeI and SacI restriction sites (Tiwari et al., 2004; Tian et al., 2015). The *35S:PIP2* and *35S:PIP3* fragments in the *pUC19-35S:PIP2* and *pUC19-35S:PIP3* constructs were then digested with Pst1 and Sac1 enzymes and sub-cloned into the binary vector *pPZP211* (Hajdukiewicz et al., 1994). The primers used to amplify *PIP2* were 5'-CAACATATGATGATGAACAAAAACGTTCTG-3' and 5'-CAAGAGCTCTTAGTGGCCCGGTCCG-3', to amplify *PIP3* were, 5'-CAACATATGATGATGAACAAAGTTGTTTTGG-3', and, 5'-CAAGAGCTCTTAGTGACCGGGTCCACTC-3'.

To generate CRISPR/Cas9 constructs for gene editing of *PIP2* and *PIP3*, exon sequences of *PIP2* and *PIP3* were evaluated on CRISPRscan^1^ for potential target sequences. Target specificity was then assessed on Cas-OFFinder.^2^ The cas9 targeted sequences selected for *PIP2* were 5'-GTTCTTCATGTTGATTGGTT(CGG)-3' and 5'-GCTTGGTCTAACAAAGACCG(AGG)-3', for *PIP3* were 5'-GTGGTGGAGGCTCGTCCTTT(GGG)-3' and 5'-GAAGGCTGAAGAGAACCTCG(TGG)-3'. The target sequences were inserted into the *pHEE-FT* vector (Cheng et al., 2019). The primer used to generate CRISPR/Cas9 constructs for editing *PIP2* were DT1-BsF (*PIP2*), 5'-ATATATGGTCTCGATTGTTCTTCATGTTGATTGGTTGTT-3', DT1-F0 (*PIP2*), 5'- TGTTCTTCATGTTGATTGGTTGTTTTAGAGCTAGAAATAGC-3', DT2-R0 (*PIP2*), 5'-AACCGGTCTTTGTTAGACCAAGCAATCTCTTAGTCGACTCTAC-3, DT2-BsR (*PIP2*), 5'- ATTATTGGTCTCGAAACCGGTCTTTGTTAGACCAAGCAA-3'; for editing *PIP3* were DT1-BsF (*PIP3*), 5'-ATATATGGTCTCGATTGTGGTGGAGGCTCGTCCTTTGTT-3',

DT1-F0 (*PIP3*), 5'-TGTGGTGGAGGCTCGTCCTTTGTTTTAGAGCTAGAAATAGC-3',

DT2-R0 (*PIP3*), 5'-AACCGAGGTTCTCTTCAGCCTTCAATCTCTTAGTCGACTCTAC-3', DT2-BsR (*PIP3*), 5'-ATTATTGGTCTCGAAACCGAGGTTCTCTTCAGCCTTCAA-3'; for editing both *PIP2* and *PIP3* were DT1-BsF (*PIP2&PIP3*), 5'-ATATATGGTCTCGATTGTGGTGGAGGCTCGTCCTTTGTT-3',

DT1-F0 (*PIP2&PIP3*), 5'-TGTGGTGGAGGCTCGTCCTTTGTTTTAGAGCTAGAAATAGC-3', DT2-R0 (*PIP2&PIP3*), 5' AACAACCAATCAACATGAAGAACAATCTCTTAGTCGACTCTAC-3',

DT2-BsR (*PIP2&PIP3*), 5'-ATTATTGGTCTCGAAACAACCAATCAACATGAAGAACAA -3'. *U6-26-IDF* and *U6-29-IDR* primers used for colony PCR and sequencing of the CRISPR/Cas9 constructs have been described previously (Chen et al., 2019a).

### Plants Transformation, Transgenic Plants Selection, and Cas9-Free Mutant Isolation

To generate overexpress plants and *Cas9* free mutants, about 1-month-old Col wild type plants with several mature flowers were transformed with *pPZP211-35S:PIP2, pPZP211-35S:PIP3*, and the CRISPR/Cas9 constructs respectively, *via Agrobacterium tumefaciens* (GV3101) mediated floral dip method (Clough and Bent, 1998).

The *35S:PIP2* and *35S:PIP3* overexpression plants were selected as described previously (Wang et al., 2020). Multiple homozygous lines were obtained and two lines with high expression levels of *PIP2* and *PIP3*, respectively were used for the experiments.

Gene edited mutants were selected by germinating the T1 seeds on 1/2 MS plates containing 50 μg/ml Kanamycin and 100 μg/ml Carbenicillin, examining gene editing status in the early flowering plants by amplifying and sequencing the genomic sequence *PIP2* and *PIP3*, respectively, and then selecting homozygous mutants from normal flowering T2 plants. The absent of T-DNA insertion in the homozygous mutants were confirmed by PCR amplification of *Cas9* gene fragment as described previously (Cheng et al., 2019).

### DNA Isolation and PCR

To check the editing status of *PIP2* and *PIP3*, DNA was isolated from the leaves of T1 or T2 transgenic plants. The extracted DNA was used as a template for PCR amplification using genomic primers specific to *PIP2* and *PIP3*, respectively. To obtain Cas9 free mutant plants, DNA was isolated from the leaves of T2 progeny of the edited T1 plants, and used as template for PCR amplification using *Cas9* specific primer. The primers used for PCR amplification of *Cas9* gene have been described previously (Chen et al., 2019a).

### Primary Root Length Assays

Primary root length of the Col wild type, the *35S:PIP2* and *35S:PIP3* transgenic plant seedlings, and the *pip2*, *pip3*, *pip2 pip3* mutant seedlings were assayed as described previously (Wang et al., 2019). For each line, 21–25 seedlings were used for the experiments.

### Hypocotyl Length Assays

Seeds of the Col wild type, the *35S:PIP2* and *35S:PIP3* transgenic plants, and the *pip2*, *pip3*, and *pip2 pip3* mutants were sterilized and sown on 1/2 MS plates, kept at 4°C in the dark for 2 days, and then moved to a growth room with dim light (~60 μmol m^−2^ s ^−1^). Four-day-old seedlings were used for hypocotyl length assays as reported previously (Wang et al., 2007; Gao et al., 2008). Pictures were taken by using a Nikon digital camera, and the hypocotyl length was calculated by using Image J software. For each line, 29–42 seedlings were used for the experiments.

### Hypocotyl Cell Number and Cell Length Assays

Hypocotyl cell number and cell length were measured as described previously with some modifications (Scheres et al., 1994; Wang et al., 2007; Gao et al., 2008; Qu et al., 2017). In brief, 4-day-old dim light-grown seedlings of the Col wild type, the *35S:PIP2* and *35S:PIP3* transgenic plants, and the *pip2*, *pip3*, and *pip2 pip3* mutants were mounted in a film of water on a glass slide and covered with a cover slip to prevent dehydration. Cell number was counted under an OLYMPUS BX53 microscope, at the distance between the top of the root hairs around the collet, and the base of the “V” made by the petioles of the cotyledon (Scheres et al., 1994). The second row cells from the top to the base of the hypocotyls epidermis in longitudinal direction were used for cell length measurement (Qu et al., 2017). Pictures were taken under an OLYMPUS BX53 microscope, and cell length was measured by using Image J. For each line, 22–29 seedlings were used for the experiments.

## Results

### *PIP2* Is an Auxin Response Genes

It has been previously reported that the expression of some plant peptide hormone genes including *PLS* and *RGF*/*CLEL*/*CLV* was regulated by auxin (Casson et al., 2002; Chilley et al., 2006; Meng et al., 2012b; Whitford et al., 2012; Guo et al., 2015). The *PIPLs* peptide hormone gene *PIPL3* has recently been shown to regulate lateral root initiation in *Arabidopsis*, a process controlled by auxin (Toyokura et al., 2019), inducing a cross talk between PIP peptide hormone and auxin.

**Figure 1:**
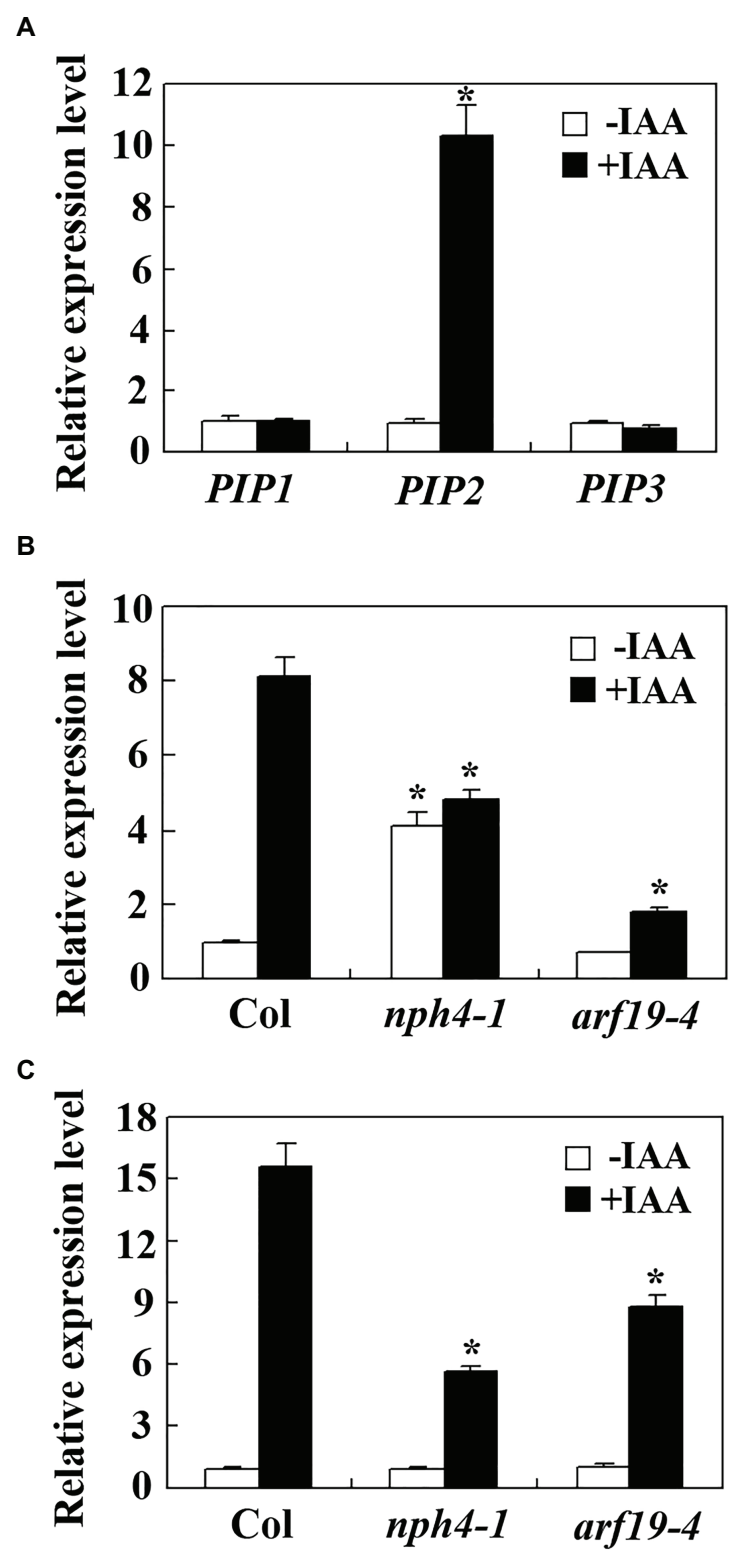
*PIP2* is an auxin response gene. **(A)** Expression of PAMP-INDUCED SECRETED PEPTIDES (*PIPs*) in response to auxin treatment. Ten-days-old Col wild type seedlings were treated with 10 μM INDOLE-3-ACETIC ACID (IAA) for 4 h, total RNA was isolated and quantitative RT-PCR (qRT-PCR) was used to examine the expression of *PIPs*. Expression of *ACTIN2* (*ACT2*) was used as an inner control, and the expression level of corresponding *PIP* genes in the control seedlings was set as 1. Data represent mean ± SD of three repeats. *significantly different from absent of IAA (student’s *t* test, *p* < 0.001). Expression of *PIP2* **(B)** and *IAA19* **(C)** in the *nph4-1* and *arf19-4* mutants in response to auxin treatment. Ten-day-old Col wild type, *nph4-1* and *arf19-4* mutant seedlings were treated with 10 μM IAA for 4 h. Total RNA was isolated and qRT-PCR was used to examine the expression of *PIP2* or *IAA19*. Expression of *ACT2* was used as an inner control, and the expression level of *PIP2* or *IAA19* in control seedlings of the Col wild type was set as 1. Data represent mean ± SD of three repeats. *significantly different from the corresponding expression level in the Col wild type seedlings (student’s *t*-test, *p* < 0.001). The experiments were repeated three times with similar results.

To examine if PIP peptide hormones may be involved in the regulation of auxin regulated plant growth and development. We first examined the expression of *PIP* genes including *PIP1*, *PIP2*, and *PIP3* in response to auxin. Seedlings of the Col wild type *Arabidopsis* were treated with IAA for 4 h and qRT-PCR was used to examine the expression of the *PIP* genes. As shown in Figure 1A, the expression level of *PIP2* increased about 10 folds in response to auxin treatment, whereas the expression level of *PIP1* and *PIP3* remained largely unchanged, suggest that *PIP2* is an auxin response gene, but *PIP1* and *PIP3* are not.

It has been shown that five of the ARFs, including ARF5, ARF6, ARF7, ARF8, and ARF19 are activators that positively regulating the expression of some auxin response genes (Tiwari et al., 2003; Wang et al., 2005), to examine if they may involve in the regulation of *PIP2*, we examine auxin response of *PIP2* in *nph4-1/arf7* and *arf19-4*, two ARF activator gene mutants in hand by using qRT-PCR. We found that the auxin response of *PIP2* was decreased in both *nph4-1* and *arf19-4* mutants (Figure 1B), suggest that ARF7 and ARF19 may regulate the expression of *PIP2*. To our surprise, we found that the basal expression level of *PIP2*, i.e., in the absence of auxin was increased about 4-fold in the *nph4-1* mutant (Figure 1B). As a control, auxin response of *IAA19* was reduced in the *nhp4-1* and *arf19* mutants, but their basal expression levels remained largely unchanged in both mutants (Figure 1C), a result similar as reported previously (Wang et al., 2005).

### Generation of *PIP2* Gene Mutants by CRISPR-Cas9 Gene Editing

**Figure 2:**
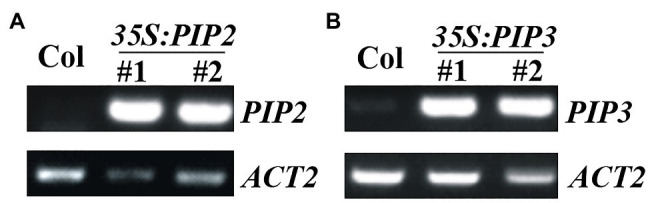
Expression of *PIP2* and *PIP3* in the *35S:PIP2* and *35S:PIP3* transgenic plants. **(A)** Expression of *PIP2* in the *35S:PIP2* transgenic plants. **(B)** Expression of *PIP3* in the *35S:PIP3* transgenic plants. Total RNA was isolated from 10-day-old homozygous transgenic plants and RT-PCR was used to examine the expression of *PIP2* or *PIP3*. Expression of *ACT2* was used as a control.

To examine the functions of PIP2, we generated plants overexpressing *PIP2*, and gene edited mutants of *PIP2* gene *via* CRISPR/Cas9 mediated gene editing. Overexpression plants were generated by transforming Col wild type *Arabidopsis* with *pPZP211-35S:PIP2* construct, selecting homozygous plants in T3 generation, and examining the expression level of *PIP2* in the homozygous transgenic plants (Figure 2A). We also generated *PIP3* overexpression plants (Figure 2B), in order to compare the functions of auxin responsive and non-responsive *PIP* genes. Two independent lines with similar expression levels of *PIP* genes were selected for further experiments.

Gene edited mutants of *PIP2* gene was generated by transforming Col wild type *Arabidopsis* with *PIP2* targeting CRISPR/Cas9 construct generated by using a *pHEE-FT* vector (Cheng et al., 2019), checking gene editing status in early flowering T1 plants, selecting Cas9-free homozygous mutants in normal flowering T2 generations. For comparison, we generated gene edited mutant for *PIP3* gene by transforming Col wild type *Arabidopsis* with *PIP3* targeting CRISPR/Cas9 construct, as well as mutants with both *PIP2* and *PIP3* genes were edited by transforming Col wild type *Arabidopsis* with CRISPR/Cas9 construct targeting both *PIP2* and *PIP3*.

**Figure 3:**
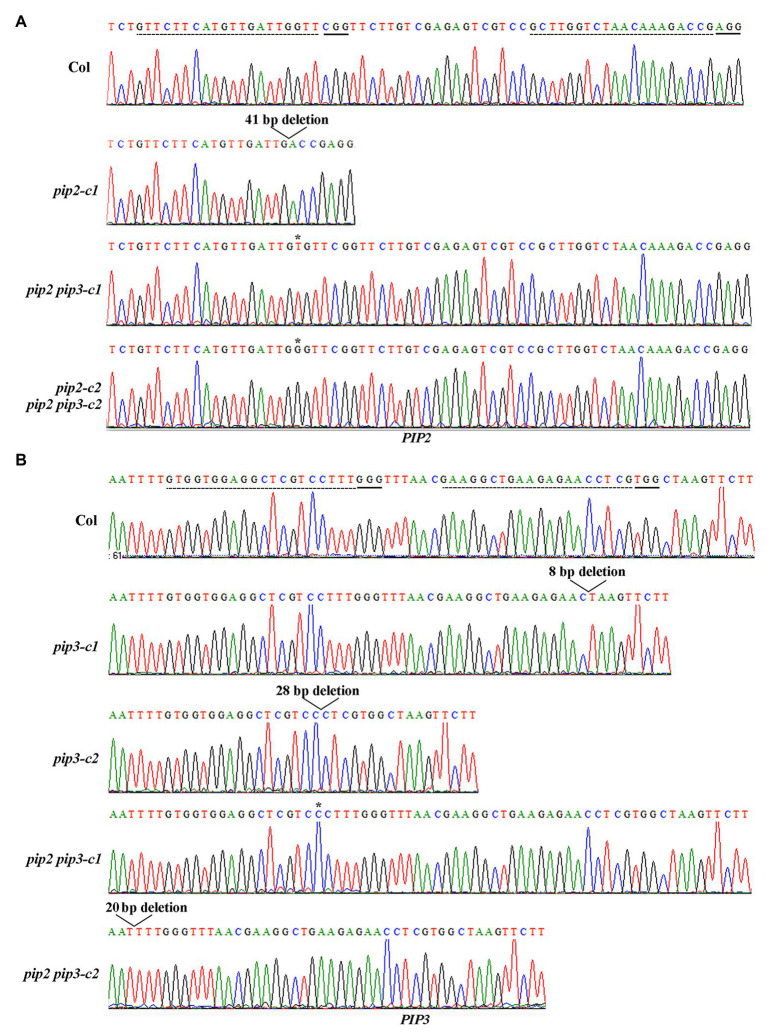
Generation of the *pip2*, *pip3*, and *pip2 pip3* mutants by CRISPR/Cas9 gene editing. **(A)** Editing status of *PIP2* in the *pip2* and *pip2 pip3* mutants. **(B)** Editing status of *PIP3* in the *pip3* and *pip2 pip3* mutants. DNA was isolated from leaves collected from early bolting T1 plants or normal bolting T2 plants, and PCR was used to amplify the coding sequence of *PIP2* and/or *PIP3*. The PCR products were recovered and sequenced, and sequencing results were compared with genome sequence of *PIP2* or *PIP3* to check the editing status. Dash lines indicate the target sequences, and solid lines indicate the PAM sites.

**Figure 4:**
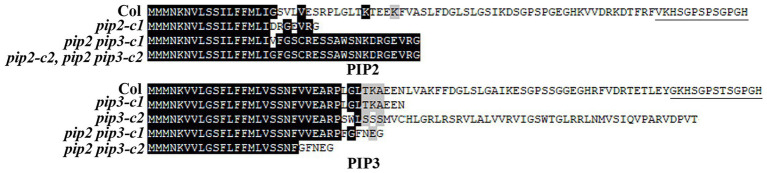
Amino acid alignment of PIP2 and PIP3 in the Col wild type and the *pip2*, *pip3* and *pip2 pip3* mutants. The open-reading frame (ORF) of *PIP2* and *PIP3* sequences in the *pip2* and *pip2 pip3* mutants were identified by using ORFfinder (https://www.ncbi.nlm.nih.gov/orffinder/), and corresponding amino acid sequences were used for alignment with the amino acid sequences of PIP2 and PIP3, respectively. Under lines indicate the mature PIP2 and PIP3 peptides.

Two independent single mutants for *PIP2* and *PIP3* genes respectively, i.e., *pip2-c1*, *pip2-c2*, *pip3-c1*, and *pip3-c2*, and two independent double mutants, i.e., *pip2 pip3-c1*, *pip2 pip3-c2* were obtained and used for the experiments. In the *pip2* mutants, either a single nucleotide insertion or a small fragment deletion was occurred (Figure 3A). For both the *pip3* mutants, a small fragment deletion was occurred (Figure 3B). Whereas in the *pip2 pip3* double mutants, a single nucleotide insertion was occurred for *PIP2* (Figure 3A), and either a single nucleotide insertion or a small fragment deletion was occurred for *PIP3* (Figure 3B). All the nucleotide insertion or small fragment deletion led to amino substitution and premature stop of the ORF, as a result, the predicated amino acid sequences for *PIP2* and *PIP3* genes in the single and double mutants leak the amino acids of the mature PIP2 and PIP3 peptides (Figure 4).

### PIP2 and PIP3 Affect Root Elongation in *Arabidopsis* Seedlings

**Figure 5:**
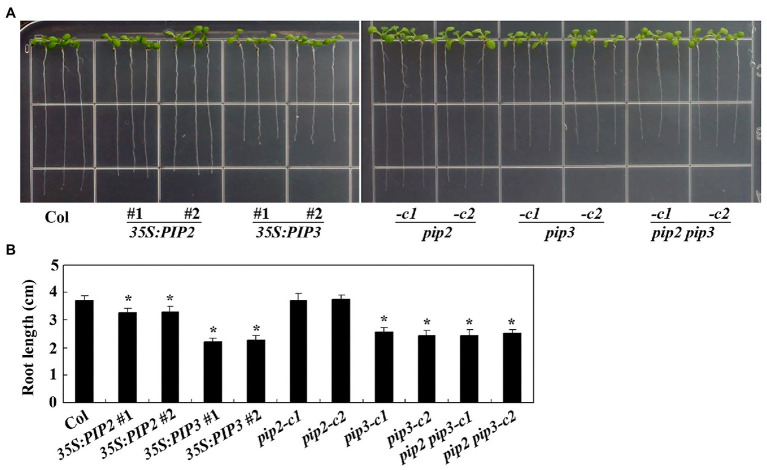
PIP2 and PIP3 affect root elongation. **(A)** Root elongation in seedlings of the Col wild type, the *35S:PIP2* and *35S:PIP3* transgenic plants, and the *pip2*, *pip3* and *pip2 pip3* mutants. Seeds were sterilized and plated on 1/2 Murashige and Skoog (MS) plates, kept at 4°C and in darkness for 2 days before transferred to a growth room and grown vertically for 8 days. Pictures were taken by using a digital camera. **(B)** Root length in seedlings of the Col wild type, the *35S:PIP2* and *35S:PIP3* transgenic plants, and the *pip2*, *pip3*, and *pip2 pip3* mutants. Root length of 8-day-old seedlings was measured. Data represent the mean ± SD of 21–25 seedlings. *significantly different from that of the Col wild type seedlings (student’s *t*-test, *p* < 0.001). The experiments were repeated three times with similar results.

As regulating root elongation is one of the characterized functions of auxin (Rehman et al., 2007), we examine the possible roles of PIP2 in root elongation by using the overexpression plants and gene edited mutants generated. Sterilized seeds of the Col wild type, the *35S:PIP2* transgenic plants and the *pip2* mutants were plated on 1/2 MS plates, and grown vertically for root elongation observation. As shown in Figure 5A, the *35S:PIP2* transgenic plant seedlings produced short roots when compared with the Col wild type seedlings, whereas that in the *pip2* mutant seedlings remained largely unchanged. Quantitative analysis showed that the root length of the *35S:PIP2* transgenic plant seedlings were about 90% of the Col wild type (Figure 5B). On the other hand, the transgenic plant seedlings expressing *PIP3*, the non-auxin responsive *PIP* gene, produced much shorter roots when compared with that in the Col wild type seedlings, and the root length in the *pip3* mutant seedlings was also reduced (Figure 5A). The root length in both the *35S:PIP3* transgenic plant seedlings and the *pip3* mutant seedlings was about 60% of the Col wild type seedlings (Figure 5B). We also found that root length in the *pip2 pip3* double mutant seedlings is largely similar to that in the *pip3* single mutant seedlings (Figure 5).

### PIP2 Affects Hypocotyl Elongation

Having shown that PIP2 is involved in the regulation of root elongation, we want further examine the cellular basis of PIP2 in regulating root elongation, i.e., if PIP2 may affects cell division and cell elongation. Considering that cell division and cell elongation in root may vary at different development stages, we sought to examine cell division and cell elongation of epidermis cells in hypocotyls, where the number of epidermis cells is pre-determined during embryogenesis (Gendreau et al., 1997), and has been shown to be a reliable and robust system for simultaneously detect defects in cell division and cell elongation (Ullah et al., 2001, 2003; Gao et al., 2008).

**Figure 6:**
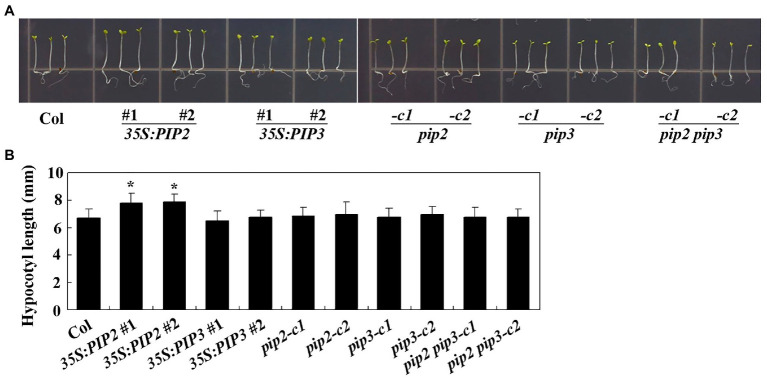
PIP2 affects hypocotyl elongation. **(A)** Hypocotyl elongation in seedlings of the Col wild type, the *35S:PIP2* and *35S:PIP3* transgenic plants, and the *pip2*, *pip3*, and *pip2 pip3* mutants. Seeds were sterilized and plated on 1/2 MS plates, kept at 4°C and in darkness for 2 days before transferred to a growth room and grown under dim light. Pictures were taken 4 days after the transfer by using a digital camera. **(B)** Hypocotyl length in seedlings of the Col wild type, the *35S:PIP2* and *35S:PIP3* transgenic plants, and the *pip2*, *pip3*, and *pip2 pip3* mutants. Hypocotyl length of 4-day-old seedlings grown under dim light were measured. Data represent the mean ± SD of 29–42 seedlings. *significantly different from that of the Col wild type seedlings (student’s *t*-test, *p* < 0.001). The experiments were repeated three times with similar results.

To examine the effects of PIP2 in cell division and cell elongation, we first examined hypocotyl elongation in the Col wild type, the *35S:PIP2* transgenic plant and the *pip2* mutant seedlings. Sterilized seeds the Col wild type, the *35S:PIP2* transgenic plants and the *pip2* mutants were plated on 1/2 MS plates grown vertically under dim light for hypocotyl length assays. We found that, unlike that observed in root elongation, seedlings of the *35S:PIP2* transgenic plant produced longer hypocotyls (Figure 6A), i.e., an ~15% longer compared with the Col wild type seedlings (Figure 6B), whereas that in the *pip2* mutant seedlings remained similar to the Col wild type (Figure 6). On the other hand, although root length was affected in both the *35S:PIP3* transgenic plant and the *pip3* mutant seedlings (Figure 6), the hypocotyl length in the seedlings of these plants is largely unaffected, and the hypocotyl length in the *pip2 pip3* double mutants is also indistinguishable from the Col wild type seedlings (Figure 6).

### PIP2 Affects Cell Division and Elongation in Hypocotyls

**Figure 7:**
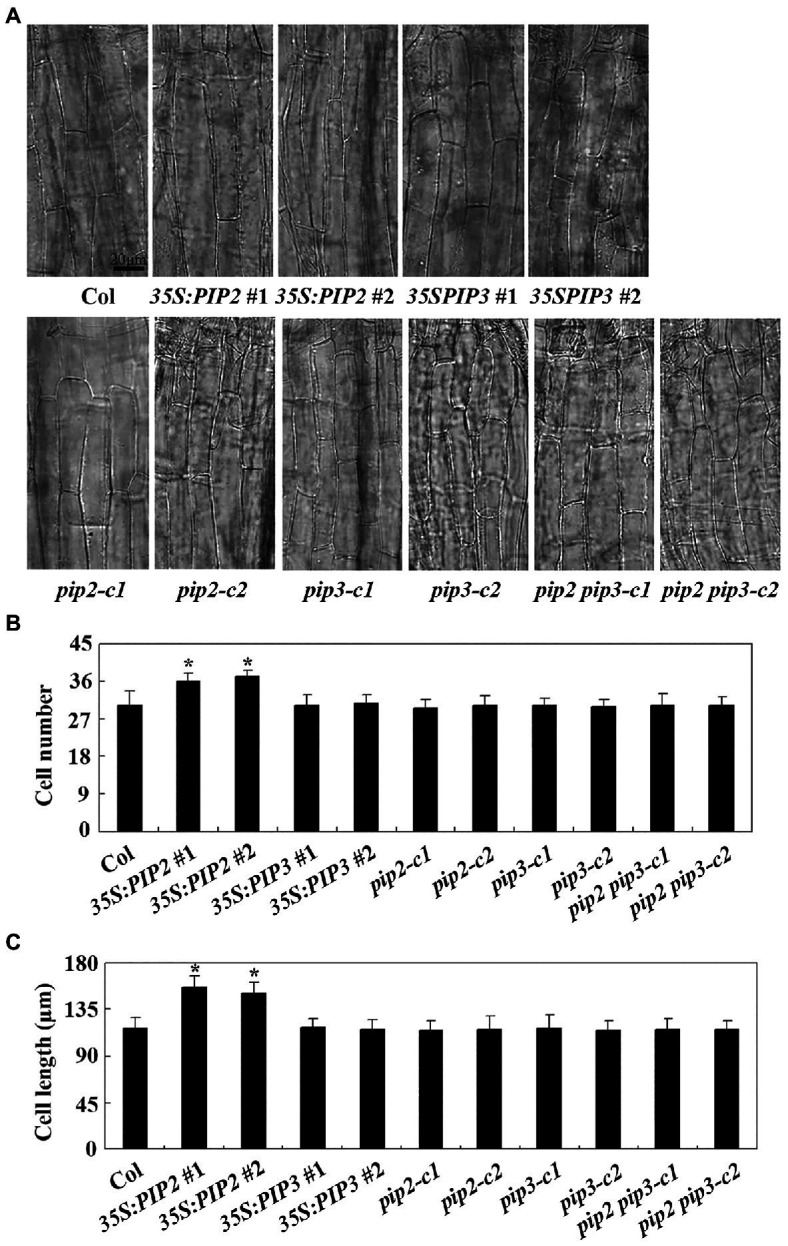
PIP2 affects cell prolification and cell elongation in hypocotyls. **(A)** Hypocotyl cells in seedlings of the Col wild type, the *35S:PIP2* and *35S:PIP3* transgenic plants, and the *pip2*, *pip3*, and *pip2 pip3* mutants. Four-day-old dim light-grown seedlings were fixed and pictures were taken under an OLYMPUS BX53 microscope. **(B)** Number of hypocotyl cell in seedlings of the Col wild type, the *35S:PIP2* and *35S:PIP3* transgenic plants, and the *pip2*, *pip3* and *pip2 pip3* mutants. Cell number of a single cell line of the 4-day-old seedlings grown under dim light was counted under an OLYMPUS BX53 microscope. Data represent the mean ± SD of 10–12 seedlings. *significantly different from that of the Col wild type seedlings (student’s *t*-test, *p* < 0.001). The experiments were repeated three times with similar results. **(C)** Hypocotyl cell length in seedlings of the Col wild type, the *35S:PIP2* and *35S:PIP3* transgenic plants, and the *pip2*, *pip3*, and *pip2 pip3* mutants. Length of the second row cells from the top to the base of the hypocotyls epidermis in longitudinal direction were was measured by using Image J. Data represent the mean ± SD of 22–29 seedlings. *significantly different from that of the Col wild type seedlings (student’s *t*-test, *p* < 0.001). The experiments were repeated three times with similar results.

We then examined cell division and cell elongation of epidermis cells in hypocotyls of dim light grown seedlings of the Col wild type, the *35S:PIP2* transgenic plants and the *pip2* mutants. As shown in Figure 7A, the overall morphology of the epidermis cells in the *35S:PIP2* transgenic plants and the *pip2* mutant seedlings are largely indistinguishable from that in the Col wild type plants. However, quantitative analysis shows that the hypocotyls of the *35S:PIP2* transgenic plants produced more epidermis cells, i.e., ~36 cells in a single cell file in the *35S:PIP2* transgenic plant seedlings compared to ~30 cells in the Col wild type seedlings (Figure 7B). In addition, epidermis cell length in the hypocotyls of the *35S:PIP2* transgenic plant seedlings was also increased, i.e., ~150 μM in the *35S:PIP2* transgenic plant seedlings compared to ~120 in the Col wild type seedlings. Consistent with hypocotyl length, no changes in epidermis cell number and cell length were observed in hypocotyls of the *35S:PIP3* transgenic plant seedlings, the *pip2* and the *pip2* single and the *pip2 pip3* double mutant seedlings (Figure 7).

## Discussion

Accumulated experiment evidence suggest that there are cross talks between the plant hormone auxin and the plant peptide hormones. It has been shown that auxin is able to regulated the expression of some plant peptide hormone genes (Casson et al., 2002; Chilley et al., 2006; Meng et al., 2012b; Whitford et al., 2012; Yang et al., 2014), and some plant peptide hormones are able to regulate auxin transport (Casson et al., 2002; Chilley et al., 2006; Meng et al., 2012b; Whitford et al., 2012; Yang et al., 2014). Consistent with the presence of cross talks between plant hormones and plant peptide hormones, some plant peptide hormones and auxin response genes have been shown to be able to regulate the same specific aspects of plant growth and development (Casson et al., 2002; Matsuzaki et al., 2010; Meng et al., 2012a; Fernandez et al., 2013; Yang et al., 2014).

PIPL3, a member of the PIPs and PIPLs, a plant peptide hormone family identified in recent years (Hou et al., 2014; Vie et al., 2015), has recently shown to regulate lateral root formation (Toyokura, et al., 2019). In the *gLBD16-SRDX* transgenic plants, the expression of the *TOLS2pro:GUS* reporter is induced by auxin (Toyokura, et al., 2019), indicating a cross talk between PIPL3 and auxin. At least two pieces of evidence suggest that there is also cross talk between PIP2 and auxin. One is that the expression of *PIP2* was induced by auxin, and auxin induced expression of *PIP2* was reduced in ARF activator gene mutants *nph4-1* and *arf19-4* (Figure 1). Another is that both root elongation and hypocotyl elongation, two of many aspects of plant growth and development regulated by auxin (Chapman and Estelle, 2009), are affected in the *PIP2* overexpression plant seedlings (Figures 5, 6). Yet it is possible that the *PIPs* and/or *PIPLs* whose expression is not regulated by auxin may also have cross talks with auxin, as root elongation was affected in the *PIP3* overexpression plant and *pip3* mutant seedlings (Figure 5). Generation of overexpressing plants and/or gene edited mutants for *PIP2* and *PIP3* in auxin signaling mutants may able to examine directly if there is cross talk between PIPs/PIPLs and auxin in regulating root and hypocotyl elongation.

Different from that of *PIP2*, the expression levels of *PIP1* and *PIP3* remind largely unchanged in response to auxin treatment (Figure 1), suggest that other signaling pathways may also regulate the expression of *PIPs*. As a matter of fact, previously reports showed that the expression of several genes of the *PIPs* and *PIPLs* family is induced by biotic and/or abiotic stress (Hou et al., 2014; Vie et al., 2015), suggest that other plant hormones such salicylic acid and abscisc acid may regulate the expression of *PIPs* and/or *PIPLs*. Available evidence suggest that PIP1 and PIP2 play an important role in regulating plant response to biotic stresses (Hou et al., 2014; Vie et al., 2015), eventhough the expression of both *PIP2* and *PIP3* was not affected by ABA treatment (Vie et al., 2015), considering that the expression of *PIP2* and *PIP3* was affected by salt and cold (Vie et al., 2015), it is very likely that PIP2 and PIP3 may also involve in the regulation of plant response to abiotic stresses.

To our surprise, we found that root length was reduced in both *PIP3* overexpression plant and *pip3* mutant seedlings (Figure 5), indicating that right amount of PIP3 peptides may be critical for proper root elongation. We also found that both root length and hypocotyl length in the *pip3* single and the *pip2 pip3* double mutants are indistinguishable (Figures 5, 6), suggest that they may not have redundant functions in regulating root and hypocotyl elongation. However, considering that there are three *PIP* and eight *PIPL* genes in *Arabidopsis* (Hou et al., 2014; Vie et al., 2015; Toyokura, et al., 2019), we could not rule out the possibility that PIP and/or PIPL peptide hormones may function redundantly to regulate plant growth and development. It is possible that PIP2 and PIP3 may have different functions and/or functional mechanisms in regulating plant growth and development. As a matter of fact, it has been shown that PIP2 and PIP3 regulate plant biotic response in different ways, PIP2 regulates antipathogen activity by regulating the expression of some PTI-related genes, *WRKY* genes, *flg22-induced receptor-like kinase 1* (*FRK1*) and the SAR marker gene *PR-1* (Chen et al., 2019b), whereas PIP3 regulates immunity by regulating the biosynthesis and signaling of SA and JA in *Arabidopsis* (Najafi et al., 2020). On the other hand, both PIP2 and PIP3 contain two conserved SGPS motifs (Vie et al., 2015), and may able to produce two mature peptides, which may have different functions. As an example, CLAVATA3/ESR-RELATED 18 (CLE18) produces two peptides, one functions as an inhibitor of tracheal element differentiation and root growth (Ito et al., 2006), whereas the other promotes root growth (Meng et al., 2012a).

By examining cell numbers and cell length in hypocotyls, we found that PIP2 may regulate cell division as well as cell elongation (Figure 7), therefore to regulate root and hypocotyl elongation. However, further efforts are required to explore the functional mechanism of PIP2 in regulating root and hypocotyl elongation. First, it will be of interest to identify the receptors of PIP2. Both PIP1 and PIP2 regulate plant immunity, and RECEPTOR-LIKE KINASE 7 (RLK7) has been identified as a receptor of PIP1 (Hou et al., 2014). Interestingly, RLK7 is also a receptor of TOLS2/PIPL3, (Toyokura, et al., 2019), therefore it is worthwhile to examine if RLK7 may serve as a receptor of PIP2. Second, it will be of interest to examine how the expression of *PIP2* is regulated. The expression level of *PIP2* was increased in response to auxin treatment, and auxin induced expression of *PIP2* was reduced in the *nph4-1* and *arf19-4* mutants (Figure 1). Considering that ARF activators are responsible for the activation of auxin response genes (Tiwari et al., 2003; Wang et al., 2005), these results suggest that ARF activators may regulate the expression of *PIP2*, yet more experiments are required to examine if ARF activators may directly regulate the expression of *PIP2*. Considering that *TOLS2*/*PIPL3* is a direct target of LBD16, and TOLS2/PIPL3 functions through an auxin-SLR/IAA14-ARF7/19-LBD16-TOLS2/PIPL3-RLK7-PUCHI pathway to regulate lateral root founder cell formation (Toyokura, et al., 2019), it is also possible that *PIP2* is directly regulated by LBD16 or some other regulator downstream of ARF7/ARF19. Third, identification of PIP2 regulated genes may also help reveal the functional mechanisms of PIP2. It has been reported that among the four auxin-induced LR-related reporter genes (De Rybel et al., 2010), only two were induced by TOLS2/PIPL3, suggest that TOLS2/PIPL3 may induce gene expression in a pathway independent of auxin signaling (Toyokura, et al., 2019), but this cannot rule out the possibility that TOLS2/PIPL3 may play a feedback regulating role in auxin signaling. This may be also the case of PIP2. Last but not least, our genetic evidence suggest that PIP2 play a different role in regulating root and hypocotyl elongation, i.e., inhibit root elongation (Figure 5), but promote hypocotyl elongation (Figure 6), which may cause by different sensitivities of different parts of the plant in response to the peptides produced by overexpressing *PIP2*. Considering that PIPs/PIPLs are peptide hormone, and similar to overexpress *PIP1* and *PIP2* genes in plants, exogenous application of synthetic PIP1 and PIP2 peptides are able to enhanced immune responses in *Arabidopsis* (Hou et al., 2014), it is still worthwhile to example if exogenous application synthetic PIP2 and PIP3 peptides may able to, and how to regulate plant growth and development.

Nevertheless, our results show that PIP2 is an auxin response gene, and that PIP2 may regulate root and hypocotyl elongation *via* regulating cell division and cell elongation.

## Data Availability Statement

The original contributions presented in the study are included in the article/supplementary material, further inquiries can be directed to the corresponding author.

## Author Contributions

SW conceived the study. SH, WW, and SW designed the experiments and drafted the manuscript. SH, WW, SA, XW, Adnan, YC, CW, YW, NZ, HT, and SC did the experiments. XH, WW, TW, and SW analyzed the data. All authors contributed to the article and approved the submitted version.

### Conflict of Interest

The authors declare that the research was conducted in the absence of any commercial or financial relationships that could be construed as a potential conflict of interest.

## Footnotes

^1^http://www.crisprscan.org/?page=sequence

^2^http://www.rgenome.net/cas-offinder/
